# Intraventricular Medium B Treatment Benefits an Ischemic Stroke Rodent Model via Enhancement of Neurogenesis and Anti-apoptosis

**DOI:** 10.1038/s41598-020-63598-0

**Published:** 2020-04-20

**Authors:** Yun-An Chen, Yi-Chieh Tsai, Yi-Dao Chen, Der-Zen Liu, Tai-Horng Young, Li-Kai Tsai

**Affiliations:** 1 Institute of Biomedical Engineering, College of Medicine and College of Engineering, National Taiwan University, Taipei, 100 Taiwan; 2 Department of Neurology and Stroke Center, National Taiwan University Hospital and National Taiwan University College of Medicine, Taipei, 100 Taiwan; 3 Graduate Institute of Biomedical Materials and Tissue Engineering, College of Biomedical Engineering, Taipei Medical University, Taipei, 110 Taiwan

**Keywords:** Cell death in the nervous system, Neural stem cells, Stroke

## Abstract

Enhancement of endogenous neurogenesis after ischemic stroke may improve functional recovery. We previously demonstrated that medium B, which is a combination with epidermal growth factor (EGF) and fibronectin, can promote neural stem/progenitor cell (NSPC) proliferation and migration. Here, we showed that medium B promoted proliferation and migration of cultured NSPCs onto various 3-dimentional structures. When rat cortical neurons with oxygen glucose deprivation (OGD) were co-cultured with NSPCs, medium B treatment increased neuronal viability and reduced cell apoptosis. In a rat model with transient middle cerebral artery occlusion (MCAO), post-insult intraventricular medium B treatment enhanced proliferation, migration, and neuronal differentiation of NSPCs and diminished cell apoptosis in the infarct brain. In cultured post-OGD neuronal cells and the infarct brain from MCAO rats, medium B treatment increased protein levels of Bcl-xL, Bcl-2, phospho-Akt, phospho-GSK-3β, and β-catenin and decreased the cleaved caspase-3 level, which may be associated with the effects of anti-apoptosis. Notably, intraventricular medium B treatment increased neuronal density, improved motor function and reduced infarct size in MCAO rats. In summary, medium B treatment results in less neuronal death and better functional outcome in both cellular and rodent models of ischemic stroke, probably via promotion of neurogenesis and reduction of apoptosis.

## Introduction

Stroke is the leading cause of physical and mental disability all over the world^1^. While thrombolytic therapy and endovascular thrombectomy benefit only a limited group of stroke patients, recent advanced stroke investigations are focused on regenerative therapies^2,3^. Transplanting exogenous stem cells may raise ethical concerns, activate the immune system, and promote the development of tumors^4^; therefore, endogenous neural stem/progenitor cells (NSPCs) have become another important source for cell therapy^2,3^. Adult NSPCs are located mainly in the subventricular zone (SVZ) of the lateral ventricles and the subgranular zone (SGZ) of the hippocampal dentate gyrus^5^, which robustly proliferate and migrate to the infarct area and subsequent differentiate after a stroke^6–9^. Conditional ablation of mouse NSPCs reduced post-stroke motor and cognitive functional improvement, implying that stroke-induced neurogenesis should play a significant role in post-stroke functional recovery^10^. As a result, enhancement of endogenous neurogenesis may be an attractive strategy for future stroke treatment.

Although numerous preclinical studies have demonstrated that some growth factors and small molecules can promote endogenous neurogenesis after stroke, successful reports in clinical trials for stroke patients are sparse^11^. Medium B is a combination of a proliferation-promoting factor, epidermal growth factor (EGF), and an adhesion-promoting factor, fibronectin^12^. Previously, we have shown that medium B can promote the proliferation and migration of cultured NSPCs^13^. Here, we tried to apply medium B treatment to cellular and animal models of ischemic stroke. We found that medium B not only enhanced the proliferation and migration of NSPCs, but also promoted the neuronal differentiation of NSPCs and diminished neuronal apoptosis in the presence of NSPCs. Notably, intraventricular medium B treatment reduced infarct size and improved post-stroke functional recovery in a rodent model of ischemic stroke.

## Materials and Methods

### Ethical approval statement

The animal experimental procedures and protocols were approved by the National Taiwan University Institutional Laboratory Animal Care Committee and the Utilization Committee (No. 20140276) and AAALAC-accredited facility. All procedures met the requirements of the Animal Welfare Protection Act of the Department of Agriculture, Executive Yuan, Taiwan, developed in accord with the National Institutes of Health guide for the care and use of Laboratory animals (NIH Publications No. 8023, revised 1978), and the ARRIVE (Animal Research: Reporting *In Vivo* Experiments) guideline.

### Culture of neural stem/progenitor cells

NSPCs were obtained from pregnant Wistar rats (BioLASCO, Taipei, Taiwan) at the gestational age of 15 days according to a protocol previously described^13^. Briefly, embryos were removed from the rat, and the embryonic cerebral cortices were dissected out, washed, triturated, and cultured in the complete media containing Dulbecco’s Modified Eagle Media (DMEM)/F-12 (Gibco, Pascagoula, MS) with 1% N2 supplement (Gibco), 20 ng/ml basic fibroblast growth factor (bFGF; Invitrogen, Carlsbad, CA), and 1% antibiotic solution (Gibco) without serum. Cells were counted and seeded at a density of 2 × 10^6^ viable cells/cm^2^ in T75 culture flasks (Corning, Corning, NY) containing 10 ml of complete media. Cultures were incubated at 37 °C in a humidified atmosphere and 5% CO_2_ for 6 days, by which time primary neurospheres would form.

### Three-dimensional structures

Detailed procedures of the three-dimensional structural culture are shown in Supplemental Methods. In the Transwell membrane study^14^, NSPCs were added (at 200 ± 20 neurospheres/cm^2^ density) in the upper part of Transwell plates (with 8 μm pore size) (Corning) and medium B was added in the lower part. After culture for 7 days, the NSPCs on the upper side of the Transwell membrane were removed using a cotton swab. The NSPCs migrating through the pores and locating on the lower side of the membrane were then fixed for the scanning electron micrograph (SEM) experiments as previously described^15^. In the chitosan scaffold (an artificially manufactured 3-dimentional structure) and the decellularized brain studies^16^, the NSPCs were cultured on the scaffold or in the decellularized brains for 7 days and then analyzed using SEM.

### Culture of cortical neurons

Primary cortical neurons were isolated from the cortex of the embryo brain of Wistar rats (BioLASCO) at the gestational age of 17 days. The harvested cells were seeded (at 5 × 10^5^ viable cells/cm^2^ density) onto poly-D-lysine (Sigma-Aldrich, St. Louis, MO) coated plates and cultured in the neurobasal (Gibco) medium containing 2% B27 (Gibco), 2 mM L-glutamine (Sigma-Aldrich) and 25 μM L-glutamic acid, at 37 °C, 5% CO_2_; then, after one day, in neurobasal medium supplemented with 2% B27, 2 mM L-glutamine, 1 μM arabinofuranosyl cytidine (Ara-C; Sigma-Aldrich), and 1% antibiotic solution (Gibco) without serum. The primary cultured neurons were identified with immunofluorescence.

### Oxygen-glucose deprivation model

Oxygen-glucose deprivation (OGD) was used to model an ischemia-like condition *in vitro*. Cultured cortical neurons were exposed to glucose-free DMEM (Gibco) and the culture plate was placed into a hypoxia incubator (Baker, Sanford, ME) with 0.5% O_2_, 94.5% N_2_, and 5% CO_2_ at 37 °C. After an hour in the OGD, the medium was changed to normal culture neurobasal medium containing B27, 2 mM L-glutamine, and 1% antibiotic solution and plates were returned to normoxia conditions. The cultured cortical neurons following OGD were first treated with NSPCs and/or medium B for 7 days as direct treatment. Transwell co-culture system (8 μm pore size Transwell plates; Corning) was then used for indirect treatment to mimic the *in vivo* condition. For the Transwell experiment, the cortical neurons following OGD were cultured in the lower chamber and the NSPCs and/or medium B (10 ng/mL EGF (Invitrogen) and 1 µg/mL fibronectin (Sigma)), EGF (10 ng/mL), or fibronectin (1 µg/mL) were added in the upper part of the Transwell inserts. After 7 days, cell viability was assessed using morphological assessment by light microscopy (number of cells per 0.33 mm^2^) as well as the AlamarBlue Cell Viability Reagent assay (Invitrogen) to analyze the neuronal number.

### Middle cerebral artery occlusion model

The animal experiment for middle cerebral artery occlusion (MCAO) followed the protocols as described previously with modification^17,18^. Briefly, male Wistar rats (270–350 g, BioLASCO) were anesthetized with an intraperitoneal injection of a mixture of 20.8 mg/kg Zoletil (Virbac, Carros, France) and 8.3 mg/ kg xylazine (Sigma). A monofilament nylon wire (RWD Life Science, San Diego, CA) was inserted from the right common carotid artery into the right internal carotid artery and then to the circle of Willis to occlude the origin of the right middle cerebral artery. A heating pad was placed under the rat during the surgery to maintain the body (rectal) temperature at 37 ± 0.5 °C. An hour after MCAO, the monofilament nylon wire was withdrawn for cerebral reperfusion. Sham-operated rats underwent the same procedure without insertion of the monofilament nylon wire.

Post-MCAO intraventricular medium B treatment was achieved using an Alzet osmotic minipump (infusion at a speed of 1 µl/h for 7 days; Alzet, Palo Alto, CA) that contained 100 ng/mL EGF and 10 µg/mL fibronectin. One day after MCAO, rats were anesthetized and placed in a stereotaxic apparatus with bregma and lambda in the same horizontal plane. A heating pad was used to maintain the body temperature at 37 ± 0.5 °C. A midline skin incision was made and a stainless steel cannula (28 gauge) was implanted in the right lateral ventricle (reference to bregma: anteroposterior = −0.8 mm, lateral = −1.5 mm, depth = 3.5 mm)^19^ and connected to an osmotic mini-pump (model 2001, Alzet), which was placed subcutaneously on the back of the rat. As a result, medium B would continuously infuse into the right lateral ventricle one day after MCAO for 7 days.

### Immunocytochemistry and immunohistochemistry

The density of cultured neurons were analyzed by immunocytochemistry using antibody against Ki-67 (a proliferation marker), nestin (an NSPC marker), and microtubule-associated protein 2 (MAP2, a mature neuron marker). The density or distribution of specific cells on brain slices were analyzed by immunohistochemistry using antibody against Ki-67, nestin, MAP2, glial fibrillary acidic protein (GFAP, an astrocyte marker), doublecortin (DCX, a neuroblast marker), and NeuN (a mature neuronal marker). Immunochemical detection of the Ki67 nuclear protein has been widely used to identify cells in the late G1 through M phases of the cell cycle for studying cell proliferation, including NSPC proliferation^20–22^. Detailed procedures of immunostaining are shown in Supplemental Methods.

### TUNEL staining assays

The cultured cortical neurons at day 7 after OGD and the brain sections at days 4, 7 and 15 after MCAO were stained by the terminal deoxynucleotidyl transferase dUTP nick end labeling (TUNEL) *In Situ* Cell Death Detection Kit (Sigma-Aldrich) and *In Situ* Cell Death Detection Kit, TMR red (Roche diagnostics GmbH, Mannheim, Germany) for apoptosis analysis, respectively. The brain sections were also stained for mature neuronal marker, NeuN. The images were taken by fluorescence microscope (Leica Microsystems GmbH) and confocal LSM880 microscope (Carl Zeiss AG). For cultural cells, data were expressed as the ratio of apoptotic cell (TUNEL positive) area to total cell (Hoechst positive) area in three different regions for each condition. For brain sections, data were expressed as the number of apoptotic cells (TUNEL positive) or apoptotic neurons (TUNEL and NeuN double positive) per field in three different regions for each condition.

### Western blot

The protein levels of Bcl-xL, Bcl-2, Bax, cleaved caspase-3, Akt, phospho-Akt^(Ser473)^, anti-GSK-3β, phospho-GSK-3β^(Ser9)^, β-catenin and GAPDH were analyzed using Western blot. Detailed procedures are shown in Supplemental Methods.

### TTC staining

Rats were decapitated at day 15 after MCAO and the brains were removed quickly. The sections at 2 mm thickness were cut using adult rat brain slicers (Zivic Instruments, Pittsburgh, PA) and stained with 2% solution of 2,3,5-triphenyltetrazoliumchloride (TTC) (Sigma-Aldrich) at 37 °C for 10 min to analyze the infarcted volume. The brain regions were quantified using Image J (National Institutes of Health, Bethesda, MD). The infarct area in TTC staining of each slice was obtained by subtracting the normal ipsilateral area from that of the contralateral hemisphere to reduce overestimation of the infarct area resulting from edema in the acute stage of ischemia (N = 5 for each group). We calculated the percentage of infarcted volume using the formula: Infarcted volume (%) ={[the intact contralateral hemisphere volume − (the ipsilateral hemisphere volume − the infarcted volume)]/the intact contralateral hemisphere volume} × 100%^23^.

### Behavior testing

Three behavioral tests (rotarod maintenance test, neurological severity score test, and body asymmetry test) were used to evaluate the motor function of rats as described previously^17,18^. Rats were trained for 3 consecutive days before MCAO and analyzed for 15 days after MCAO. Detailed procedures are shown in Supplemental Methods.

### Statistical analysis

The results are reported as mean ± standard error of the mean (SEM). For body weight and rotarod maintenance test, statistical significance was analyzed by one-way ANOVA followed by LSD post hoc comparisons for the normal distribution of the data as noted by the Kolmogorov-Smirnov statistic. The other data were analyzed by the Mann-Whitney test or Kruskal–Wallis test followed by the Dunn’s multiple comparisons test. A p-value of less than 0.05 was considered statistically significant. SPSS statistical software (version 10.0, IBM SPSS, IBM Analytics, Armonk, NY) was used for the statistical analyses.

## Results

### Medium B promotes NSPC proliferation and migration in 3-dimentional structures

**Figure 1:**
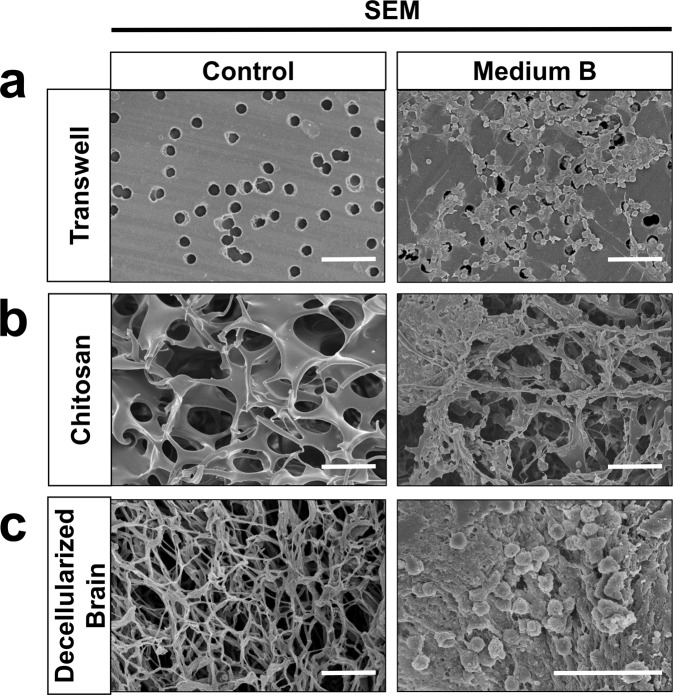
The effect of medium B on the proliferation and migration of neural stem/progenitor cells (NSPCs) on 3-dimentional structures.** (a)** In the Transwell membrane study, NSPCs were added onto the upper part of Transwell plates with or without medium B treatment. After culture for 7 days, the NSPCs migrating through the pores and locating on the lower side of the membrane were detected using scanning electron micrograph (SEM) imaging. **(b) In the chitosan scaffold study, NSPCs were cultured on the scaffold for 7 days with or without medium B treatment and then detected by** SEM imaging. **(c)** In the decellularized brain study, NSPCs were cultured on the scaffold with or without medium B treatment for 7 days and then detected by SEM imaging. Scale bar: 50 μm for **(a**,**b)**; 25 μm for **(c)**.

We previously demonstrated that medium B can enhance the proliferation and migratory capacity of NSPCs in culture^13^. Before the *in vivo* experiments, we tested the effect of medium B on NSPCs in various 3-dimentional structures, including Transwell membrane, chitosan scaffold, and decellularized brain, analyzing using SEM. With medium B treatment, NSPCs cultured on the top of Transwell membranes migrated through the small holes to the other side of the membrane, while NSPCs without medium B treatment showed a near absence of migration (Fig. 1a). In the chitosan scaffold experiment, cultured NSPCs seldom attached onto the scaffold, even so, mainly maintained the prior neurosphere structures with minimal migration. After medium B treatment, NSPCs well attached onto the scaffold and robustly migrated out of the neurospheres and diffusely distributed onto the scaffold (Fig. 1b). In the decellularized brain experiment, cultured NSPCs with no treatment or with only EGF did not attach onto the brain structure (Fig. 1c). However, medium B treatment enhanced NSPC migration and proliferation on the decellularized brain (Fig. 1c). Taken together, these results confirm that medium B promotes NSPC attachment, migration and proliferation in 3-dimentional culture systems.

### Medium B enhances post-OGD neuronal viability with existence of NSPCs

**Figure 2:**
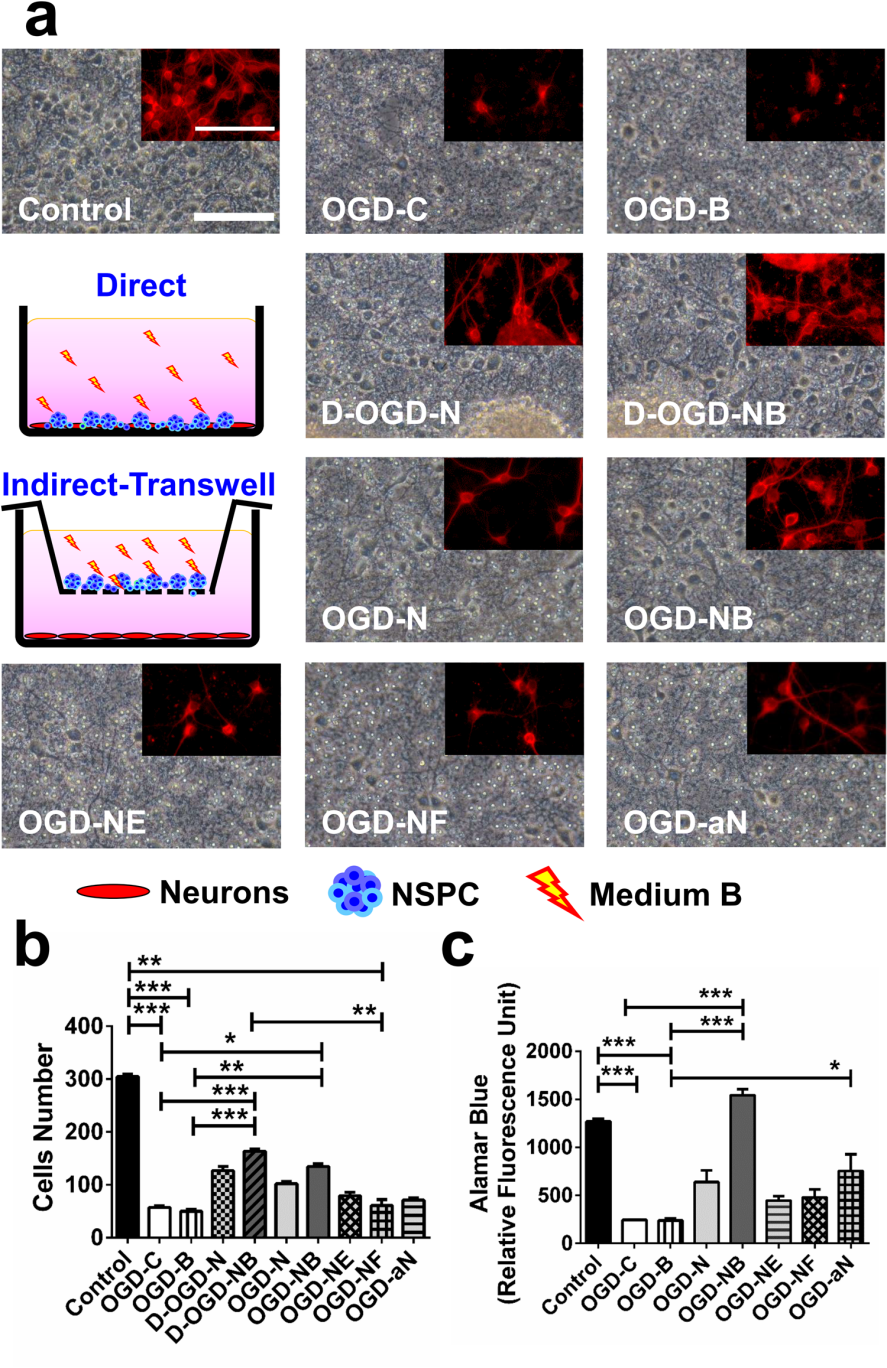
The effect of medium B on cultured neurons with oxygen glucose deprivation (OGD). **(a)** The photographs and immunocytochemistry of the anti-MAP-2 antibody (right corner subpanels, red) of cultured neurons (control), post-OGD neurons without treatment (OGD-C), and post-OGD neurons with medium B (OGD-B), neural stem/progenitor cells (NSPCs; direct, D-OGD-N; indirect, OGD-N), combined medium B and NSPC (direct, D-OGD-NB; indirect, OGD-NB), combined epidermal growth factor and NSPC (indirect, OGD-NE), combined fibronectin and NSPC (indirect, OGD-NF), and high number of NSPC (indirect, OGD-aN) treatment. For direct treatment, NSPCs were added directly into the neuronal culture medium. For indirect treatment, neurons were seeded into the lower chamber and NSPCs and/or factors were added into the upper part of the Transwell. Scale bar = 100 μm. **(b)** Neuronal viability measured by counting the number of neurons. **(c)** Neuronal viability analyzed by AlamarBlue assay, in which the groups D-OGD-N and D-OGD-NB were not included because of the interference of results by NSPCs. N = 6 for each group. *p < 0.05; **p < 0.01; ***p < 0.001.

We further set up an *in vitro* OGD model (mimicking ischemic stroke) to test the effects of medium B treatment on primary cultured neurons with transient hypoxia/hypoglycemia. Various treatments starting at one day after OGD for 7 days were tested, followed by neuronal viability analysis. The number of cultured neurons in the OGD group (OGD-C) was reduced remarkably as compared to the non-OGD group (Control) (p < 0.001) (Fig. 2a,b). While direct treatment with medium B (OGD-B) or NSPCs (D-OGD-N) (amount of 200 neurospheres/cm^2^) alone did not significantly change the post-OGD number of neurons, combined medium B and NSPC (D-OGD-NB) treatment significantly increased the number of post-OGD neurons over the untreated OGD-control (OGD-C) (163.6 ± 4.2 *vs*. 57.2 ± 2.9, p < 0.001). To further mimic the brain architecture in which the infarct region is located apart from the SVZ and SGZ, we separated cultured neurons (in the lower chamber) from NSPCs (in the upper chamber) using a Transwell co-culture system (indirect treatment). The combined medium B and NSPC (OGD-NB) treatment again increased neuronal viability as compared to the untreated OGD-control (OGD-C) (134.6 ± 4.9 *vs*. 57.2 ± 2.9, p = 0.013), but treatment by either NSPC alone (OGD-N), NSPC + EGF (OGD-NE), or NSPC + fibronectin (OGD-NF) all did not significantly change neuronal viability (Fig. 2a,b). Since medium B promotes NSPC proliferation, we also studied the effects of a high amount (800 neurospheres/cm^2^) of NSPC (OGD-aN) treatment without adding medium B, but found no increase in neuronal number after OGD.

We also used the AlamarBlue assay to measure neuronal viability via analyzing the intracellular NADH activity. Similarly, combined treatment with medium B and NSPCs (OGD-NB) in the upper chamber increased neuronal viability as compared to the untreated OGD-control (OGD-C) (1542 ± 63.5 *vs*. 244 ± 4.0, p = 0.001), but NSPC alone (amount of 200 or 800 neurospheres/cm^2^), medium B alone, NSPC + EGF, or NSPC + fibronectin treatment did not significantly change neuronal viability (Fig. 2a,c). Taken together, medium B enhanced post-OGD neuronal viability in the presence of NSPCs, while NSPC alone, medium B alone, NSPC + EGF, or NSPC + fibronectin treatment did not show any obvious benefit.

### Medium B reduced post-OGD neuronal apoptosis in the presence of NSPCs

**Figure 3:**
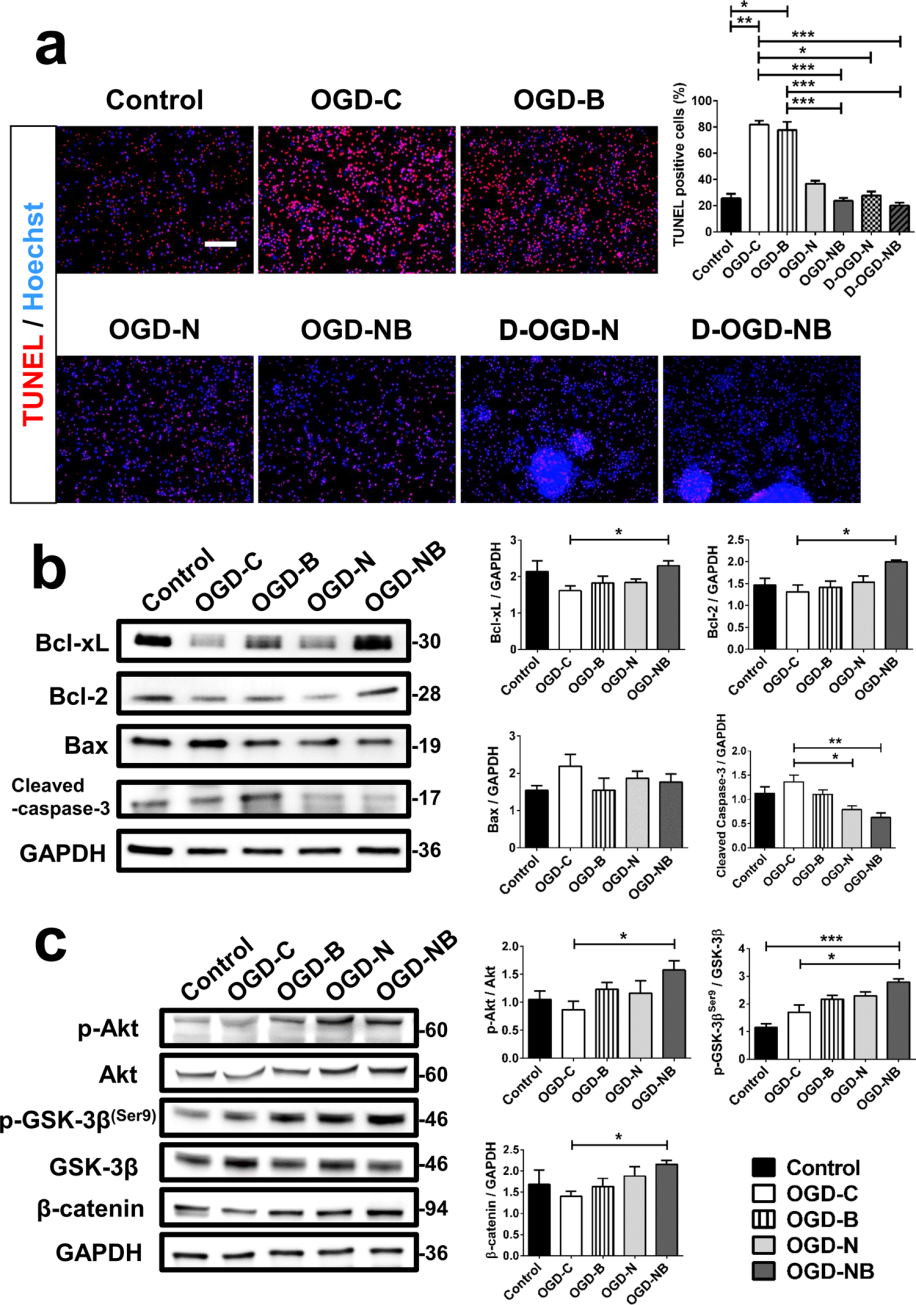
The effect of medium B on neuronal apoptosis in cultured neurons with oxygen glucose deprivation (OGD). **(a)** The terminal deoxynucleotidyl transferase dUTP nick end labeling (TUNEL) staining (red) with Hoechst (blue) nucleus staining of cultured neurons (control), post-OGD neurons without treatment (OGD-C), and post-OGD neurons with medium B (OGD-B), neural stem/progenitor cell (NSPC; direct, D-OGD-N; indirect, OGD-N) and combined medium B and NSPC (direct, D-OGD-NB; indirect, OGD-NB) treatment is shown. Scale bar = 100 μm. **(b)** Western blots of cultured neurons for Bcl-xL, Bcl-2, Bax, cleaved caspase 3, and GAPDH are shown, with analysis of the ratios of Bcl-xL, Bcl-2, Bax, and cleaved caspase 3 levels to GAPDH. Full-length blots are presented in Supplementary Fig. S3a. **(c)** Western blots of cultured neurons for phospo-Akt^(Ser473)^, Akt, phospho-GSK3-β^(Ser9)^, GSK3-β, β-catenin, and GAPDH are shown, with analysis of the ratios of: phospo-Akt level to Akt, phospho-GSK3-β level to GSK3-β, and β-catenin level to GAPDH. Full-length blots are presented in Supplementary Fig. S3b. Protein expression was obtained by normalizing band intensities to that of GAPDH analyzed with Vision Works LS software. N = 6 for each group. *p < 0.05; **p < 0.01; ***p < 0.001.

Since cultured cortical neurons cannot proliferate, we hypothesized that the high viability of post-OGD neurons with medium B and NSPC co-treatment resulted from inhibition of apoptosis. We thus used the TUNEL assay to analyze post-OGD neuronal apoptosis. Seven days after OGD, the percentage of TUNEL positive cells was higher than in the non-OGD group (p = 0.005) (Fig. 3a). Co-treatment with medium B and NSPCs with or without a transmembrane (indirect or direct treatment) both reduced the percentage of TUNEL positive cells as compared to the untreated OGD-control (20.0 ± 2.2% and 23.7 ± 2.1% *vs*. 82.0 ± 3.0%, p < 0.001). On the other hand, medium B or indirect NSPC treatment alone did not significantly decrease neuronal apoptosis.

Western blotting was then applied to analyze various apoptosis-associated factors in post-OGD neurons. Combined treatment with medium B and NSPCs significantly decreased the level of cleaved caspase 3 and increased the levels of both anti-apoptotic factors Bcl-xL and Bcl-2 (p < 0.05), as compared to the untreated OGD control in cultured neurons (Fig. 3b). The level of a proapoptotic factor, Bax, did not change after treatment. In addition, analysis of upstream anti-apoptotic factors showed that phospho-Akt, phospho-GSK-3β, and β-catenin (p < 0.05) were all increased after co-treatment with medium B and NSPCs, compared to the untreated OGD control (Fig. 3c). These findings support the assertion that medium B reduces post-OGD neuronal apoptosis in the presence of NSPCs.

### Medium B promotes NSPC proliferation and migration around the SVZ in MCAO rats

**Figure 4:**
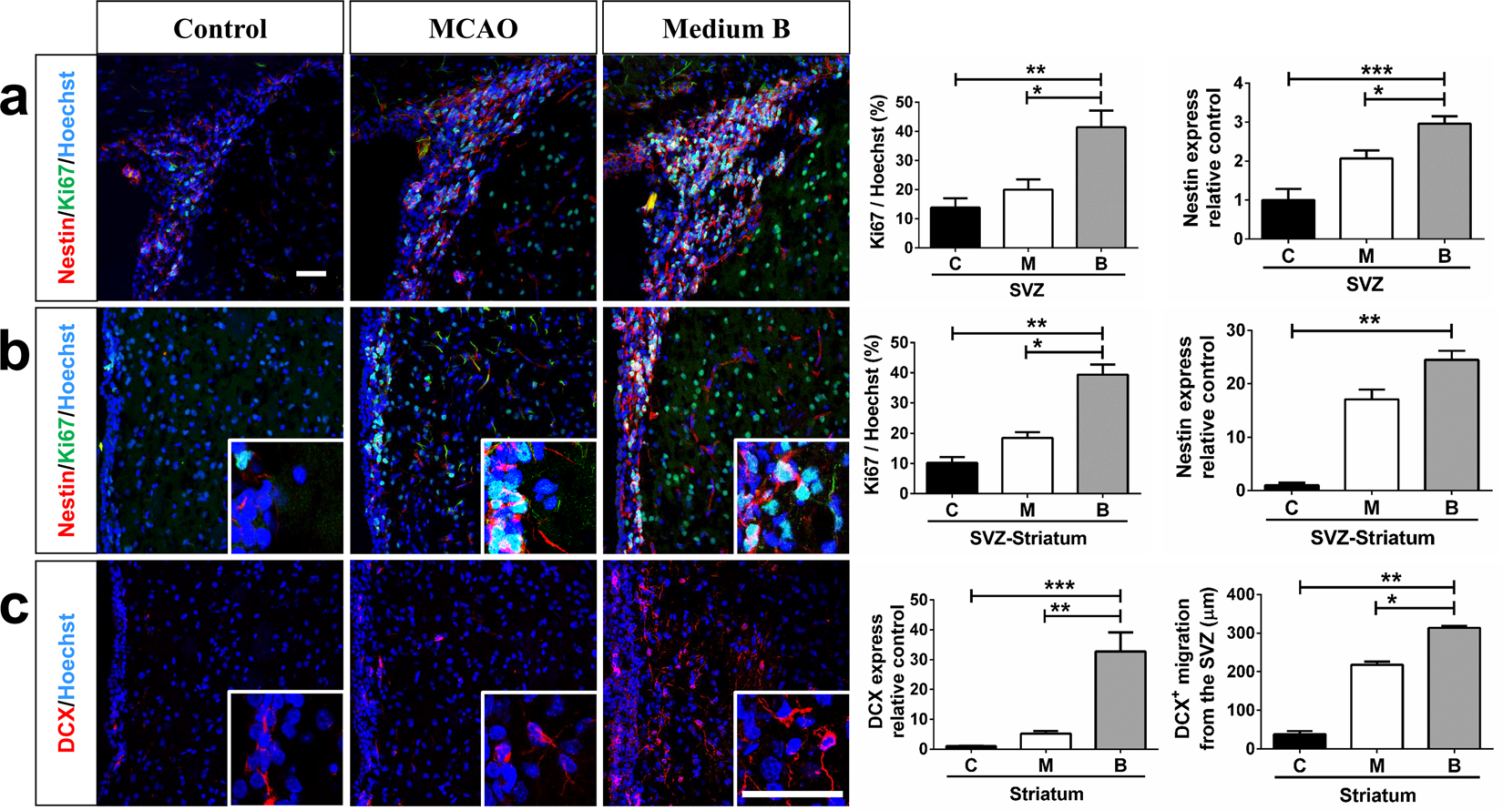
The effect of medium B on neural stem/progenitor cell (NSPC) proliferation and neuroblast migration around the subventricular zone (SVZ) in rats with middle cerebral artery occlusion (MCAO). Immunostaining with the proliferation marker Ki-67 (green), NSPC marker nestin (red), and nucleus marker Hoechst 33258 (blue) **(a)** in the SVZ and **(b)** near the SVZ of MCAO rats with and without medium B treatment and sham control rats is shown. The proliferation capacity of NSPC is presented as the percentages of Ki-67 immunoreactive cells among Hoechst positive cells or intensity of nestin immunoreactive signals relative to control. **(c)** Immunostaining with the neuroblast marker DCX (red) and Hoechst 33258 (blue) near the SVZ is shown. The migratory capacity of neuroblasts toward the striatum is presented as the density of neuroblasts located 100–300 μm away from the SVZ (DCX immunoreative signals relative to control) and the longest distance between the location of a neuroblast and the SVZ. N = 6 for each group. Scale bar = 50 μm. *p < 0.05; **p < 0.01; ***p < 0.001. C, control; M, MCAO; B, medium B.

We further investigated the effects of medium B on NSPC proliferation and migration in MCAO rats, a transient cerebral ischemic model. Fifteen days after MCAO, the number of Ki-67 immunoreactive cells in or near the ipsilateral SVZ did not differ significantly from that of sham control (Fig. 4a,b). With post-MCAO intraventricular medium B infusion, the number of Ki-67 immunoreactive cells significantly increased as compared to sham control or untreated MCAO groups, either in the SVZ (41.4 ± 5.7% *vs*. 13.9 ± 3.1% and 20.0 ± 3.5%, p < 0.05) or near the SVZ (39.4 ± 3.4% *vs*. 10.2 ± 1.9% and 18.5 ± 1.8%, p < 0.05). Medium B treatment also showed more nestin (an NSPC marker)-immunoreactive cells as compared to sham control in or near the SVZ (p < 0.05). In addition, the number of Ki67- or nestin-immunoreactive cells in the striatum and cortex was also higher in medium B-treated MCAO rats than in sham control or untreated MCAO rats (Supplemental Fig. S1).

For wide distribution of NSPCs into the infarct brain, to investigate the migratory capacity of NSPCs, we analyzed the distribution of DCX-immunoreactive cells (neuroblasts) near the ipsilateral SVZ 15 days after MCAO as previous reports^24,25^. We first analyzed the density of DCX-immunoreactive cells 100–300 μm away from the SVZ; medium B-treated MCAO rats had more such cells than sham control or untreated MCAO rats (32.7 ± 6.4 *vs*. 1 ± 0.1 and 5.2 ± 0.9, p < 0.05) (Fig. 4c). We also measured the farthest distance the neuroblast migrated from the SVZ toward the striatum. DCX-immunoreactive cells of medium B-treated MCAO rats showed longer migratory distance than those of either the sham control or untreated MCAO rats (313.6 ± 5.6 *vs*. 38.6 ± 7.6 and 217.7 ± 8.5 μm, p < 0.05) (Fig. 4c). Taken together, the results confirm that medium B treatment not only enhanced NSPC proliferation, but also promoted neuroblast migration after acute cerebral infarct.

### Medium B promoted NSPC differentiation toward neuron in MCAO rats

**Figure 5:**
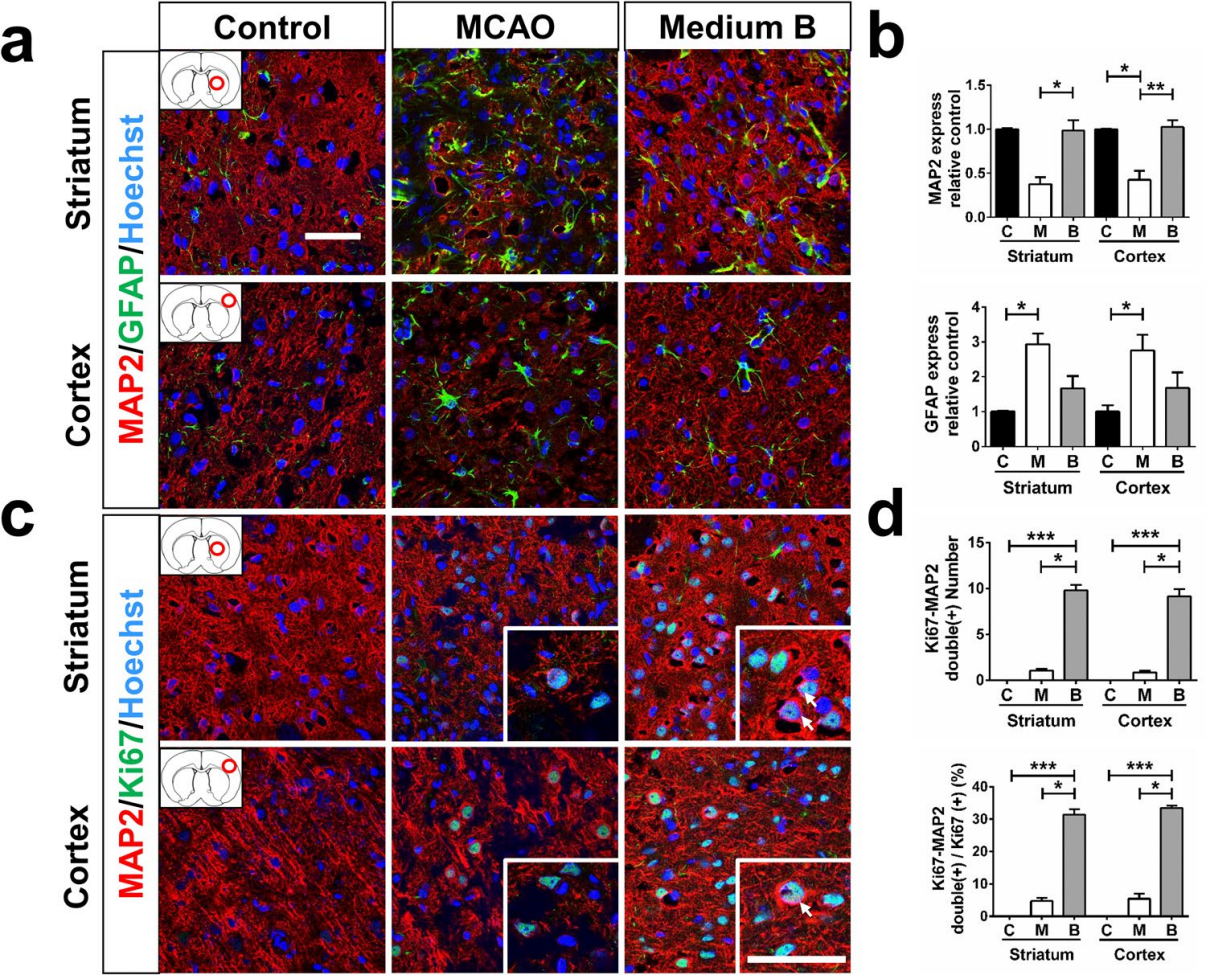
The effect of medium B on neuronal density and neurogenesis in the striatum and cortex of middle cerebral artery occlusion (MCAO) rats. **(a)** Immunostaining with the mature neuronal marker MAP-2 (red), GFAP (green), and Hoechst 33258 (blue) in the striatum and cortex of MCAO rats with and without medium B treatment and sham control rats is shown. **(b)** The neuronal or astrocyte density is presented as the intensity of MAP-2 or GFAP immunoreactive signals, respectively, relative to control. **(c)** Immunostaining with MAP-2 (red), Ki-67 (green), and Hoechst 33258 (blue) in the striatum and cortex is shown. The white arrows indicate Ki-67 and MAP-2 double immunoreactive cells. **(d)** The neurogenesis capacity is presented as the number of MAP-2 and Ki-67 double immunoreactive cells per 0.3 μm^2^ and the percentage of MAP-2 immunoreactive cells among Ki-67 immunoreactive cells. N = 6 for each group. Scale bar = 50 μm. *p < 0.05; **p < 0.01. C, control; M, MCAO; B, medium B.

Fifteen days after MCAO, the number of MAP-2 -immunoreactive cells (mature neurons) was lower in untreated MCAO than in sham control rats in the striatum and cortex (p < 0.05) (Fig. 5a,b). Medium B treatment significantly increased the density of post-MCAO MAP-2 immunoreactive cells over those in untreated rats both in the striatum (0.99 ± 0.12 *vs*. 0.38 ± 0.08, p = 0.012) and cortex (1.03 ± 0.08 *vs*. 0.43 ± 0.10, p = 0.005). In addition, while the density of GFAP-immunoreactive cells (astrocytes) was higher in untreated MCAO than in sham control rats both in the striatum and cortex (p < 0.05), the number was similar between medium B-treated MCAO and sham control rats (Fig. 5a,b).

To investigate the mechanisms of the increase in MAP-2 immunoreactive cells in infarcted brains after medium B treatment, we analyzed the effects of medium B on NSPC differentiation toward neurons in MCAO rats. The number of Ki-67 and MAP-2 double immunoreactive cells in medium B-treated MCAO rats was higher than that in untreated MCAO and sham control rats in the striatum (9.8 ± 0.6 *vs*. 1.0 ± 0.2 and 0 ± 0 per 0.3 μm^2^, p < 0.05) and cortex (9.1 ± 0.8 *vs*. 0.8 ± 0.2 and 0 ± 0 per 0.3 μm^2^, p < 0.05) (Fig. 5c,d). In addition, among the Ki-67 immunoreactive cells, the percentages of MAP-2 immunoreactive cells were also higher in medium B-treated MCAO rats than in untreated MCAO and sham control rats in the striatum (31.4 ± 1.7% *vs*. 4.7 ± 1.0% and 0 ± 0%, p < 0.05) and cortex (33.4 ± 0.8% *vs*. 5.4 ± 1.5% and 0 ± 0%, p < 0.05). In addition, the area of DCX immunoreactive cells as well as the number of DCX and Ki-67 double immunopositive cells in medium B-treated MCAO rats were higher than those in untreated MCAO rats (0.018 ± 0.001 *vs*. 0.011 ± 0.003 mm^2^ and 21.7 ± 4.9 *vs* 4.7 ± 3.7 per 0.2 mm^2^, p < 0.05) (Supplemental Fig. S2). In medium B treated MCAO rats, 31.4 ± 1.7%, 38.5 ± 6.2%, and 12.0 ± 1.0% of the Ki-67 positive cells were colocalized with MAP2, DCX, and nestin in the striatum, respectively. These results demonstrate that medium B promoted neuronal differentiation indicating neurogenesis, which probably contributed in part to the high neuronal density in infarct brains of MCAO rats.

### Medium B reduced post-MCAO neuronal apoptosis

**Figure 6:**
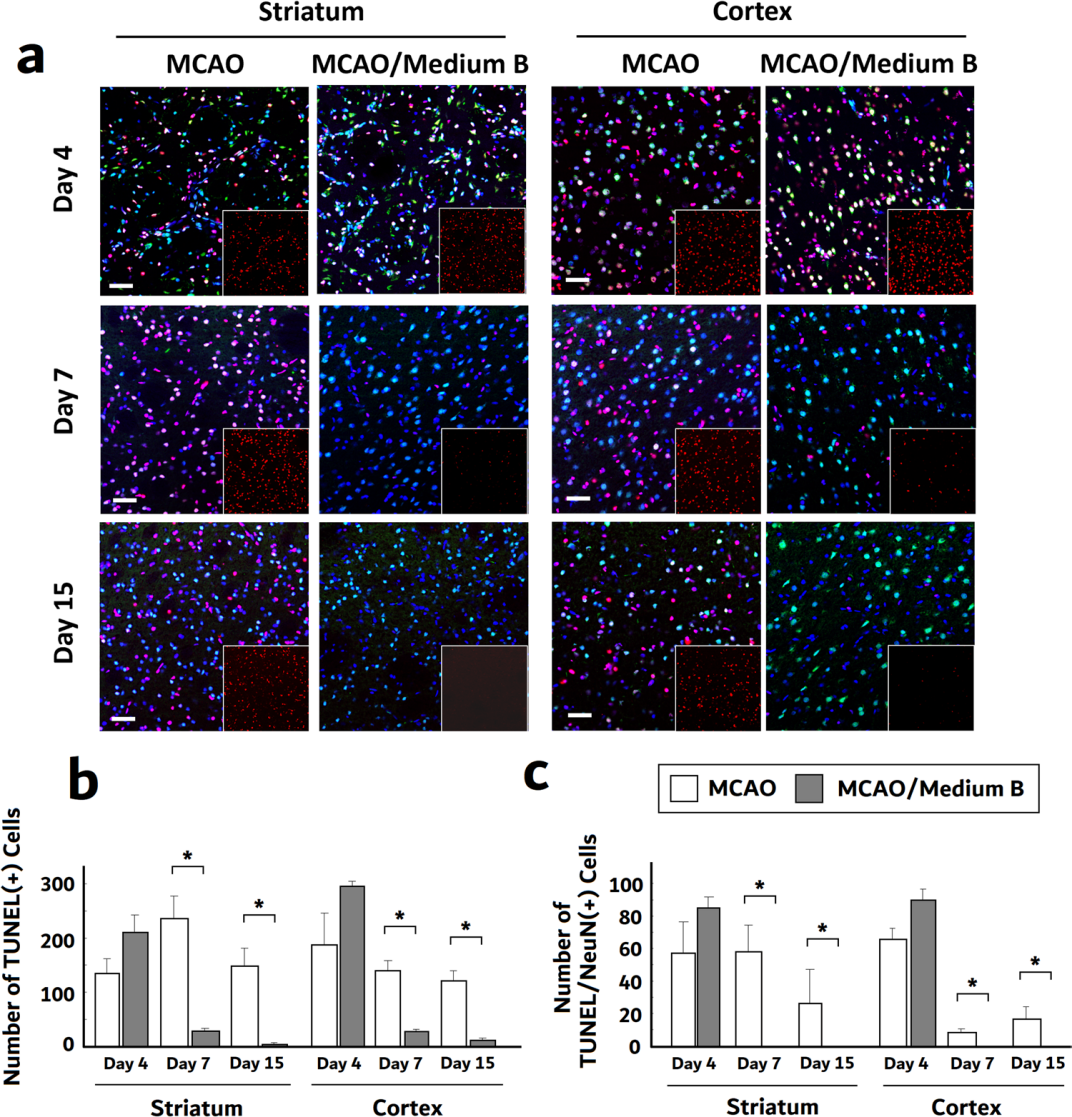
The effect of medium B on neuronal apoptosis in the striatum and cortex of middle cerebral artery occlusion (MCAO) rats. **(a)** The terminal deoxynucleotidyl transferase dUTP nick end labeling (TUNEL) staining (red) with the mature neuronal marker NeuN (green) and Hoechst 33258 (blue) nucleus staining of infarct striatum and cortex in MCAO rats with and without medium B treatment at post-MCAO days 4, 7 and 15 was shown. The right lower subpanels were TUNEL staining only. (**b)** The cell apoptosis intensity was presented as the number of TUNEL immunoreactive cells per 0.2 mm^2^. **(c)** The neuronal apoptosis intensity was presented as the number of TUNEL and NeuN double immunoreactive cells per 0.2 mm^2^. N = 3 for each group. Scale bar = 50 μm. *p < 0.05.

The increase in MAP-2 immunoreactive cells in infarcted brains after medium B treatment may also result from reduced neuronal death. To investigate the anti-apoptotic effects of medium B *in vivo* as seen in the post-OGD cultured neurons, we first used TUNEL staining to analyze the apoptotic cell density in the infarct brain. The number of TUNEL positive cells was similar between medium B-treated and untreated rats at post-MCAO day 4. However, the number of TUNEL positive cells was significantly lower in both striatum and cortex of medium B-treated than in untreated MCAO rats at post-MCAO day 7 (striatum: 30 ± 4 *vs*. 235 ± 39; cortex: 27 ± 3 *vs*. 139 ± 17 per 0.2 mm^2^, p < 0.05) and day 15 (striatum: 2 ± 0.7 *vs*. 147 ± 33; cortex: 11 ± 2 *vs*. 120 ± 19 per 0.2 mm^2^, p < 0.05) (Fig. 6a,b). To investigate the intensity of post-MCAO apoptotic neurons, we analyzed the number of TUNEL and NeuN double positive cells (Fig. 6a,c). Similarly, the number of TUNEL and NeuN double positive cells was significantly lower in both striatum and cortex of medium B-treated than in untreated MCAO rats at post-MCAO day 7 (striatum: 0 ± 0 *vs*. 58 ± 16; cortex: 0 ± 0 *vs*. 9 ± 2 per 0.2 mm^2^, p < 0.05) and day 15 (striatum: 0 ± 0 *vs*. 27 ± 20; cortex: 0 ± 0 *vs*. 17 ± 7 per 0.2 mm^2^, p < 0.05).

**Figure 7:**
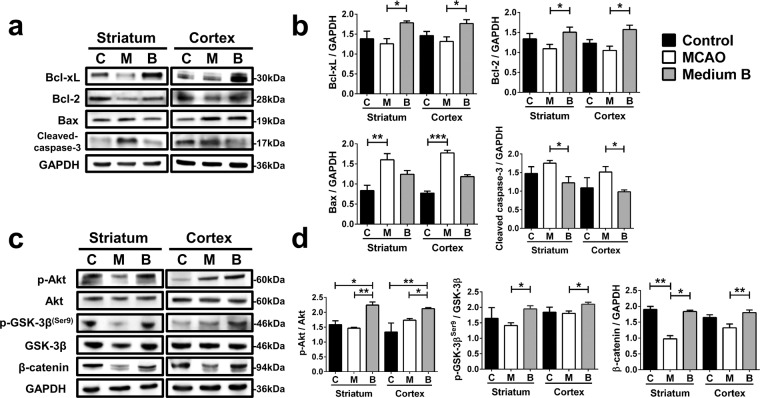
The effect of medium B on protein levels of apoptosis-related factors in the striatum and cortex of middle cerebral artery occlusion (MCAO) rats. **(a)** Western blots of homogenized brain tissues of MCAO rats with and without medium B treatment and sham control rats for Bcl-xL, Bcl-2, Bax, cleaved caspase 3, and GAPDH. Full-length blots are presented in Supplementary Fig. S4a. **(b)** Analysis of the ratio of Bcl-xL, Bcl-2, Bax, and cleaved caspase 3 levels to GAPDH. **(c)** Western blots of brain tissues to determine the presence of phospo-Akt^(Ser473)^, Akt, phospho-GSK3-β^(Ser9)^, GSK3-β, β-catenin, and GAPDH. Full-length blots are presented in Supplementary Fig. S4b. Protein expression was obtained by normalizing band intensities to that of GAPDH analyzed with Vision Works LS software. **(d)** Analysis of the ratios of phospo-Akt level to Akt, phospho-GSK3-β level to GSK3-β, and β-catenin level to GAPDH. N = 4 for each group. *p < 0.05; **p < 0.01; ***p < 0.001.C, control; M, MCAO; B, medium B.

We further analyzed apoptosis-related factors in the ipsilateral striatum and cortex of MCAO rats using Western blotting. Medium B treatment decreased the level of cleaved caspase 3 and increased the levels of both Bcl-xL and Bcl-2 (p < 0.05) over those in untreated MCAO rats (Fig. 7a,b). The level of Bax was significantly higher in untreated MCAO rats (p = 0.009) but not in medium B-treated MCAO rats as compared to sham control rats in both the striatum and cortex. In addition, the levels of phospho-Akt, phospho-GSK-3β, and β-catenin were all higher in medium B-treated than in untreated MCAO rats (Fig. 7c,d). These findings support the results from the post-OGD cultured neurons that medium B treatment can reduce cell apoptosis after acute cerebral infarction.

### Medium B facilitated functional recovery and reduced infarcted volume in MCAO rats

**Figure 8:**
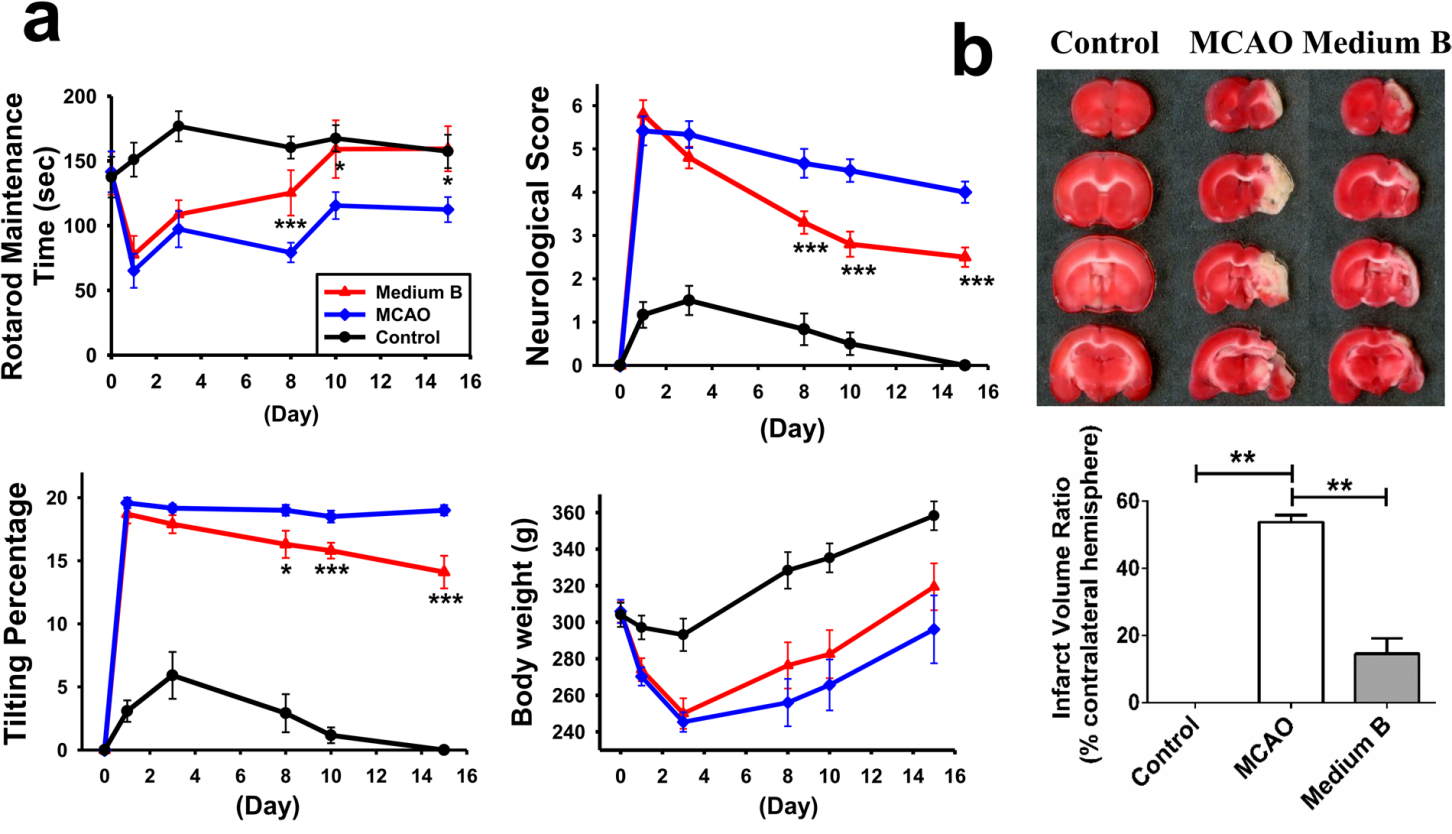
The effect of medium B on the behaviors and infarcted volume of middle cerebral artery occlusion (MCAO) rats. **(a)** The results of rotarod maintenance test, neurological severity score test, body asymmetry test, and body weight of MCAO rats with and without medium B treatment and sham control rats in 2 weeks post-MCAO. N = 10 for each group. **(b)** The 2,3,5-triphenyltetrazoliumchloride (TTC) staining for the infarcted (white) volume analysis, including measurement of the infarcted volume and calculation of the infarcted volume ratio; N = 5 for each group. *p < 0.05; **p < 0.01; ***p < 0.00.

Three behavior studies were used to evaluate neurological function within two weeks post-MCAO. One day after MCAO (before medium B treatment), the functional performances were similar between medium B-treated and untreated MCAO groups, and both performed significantly worse than sham control on the rotarod maintenance test, neurological severity score test, and body asymmetry test (p < 0.05) (Fig. 8a). Since post-MCAO day 8, the rotarod maintenance time was longer and the neurological severity score and the percentage of body asymmetry were lower in medium B-treated MCAO than in untreated MCAO rats (p < 0.05). Body weight was not significantly different between groups.

We also used TTC stain to analyze the infarcted volume at post-MCAO day 15. Sham control rats did not develop cerebral infarct, but untreated MCAO rats showed a large infarcted volume ratio (53.8 ± 2.1%) (Fig. 8b). After medium B treatment, the infarcted volume ratio reduced significantly (14.5 ± 4.6%, p = 0.002). Taken together, these results indicate that medium B treatment facilitates post-MCAO functional recovery and reduces infarcted volume in MCAO rats.

## Discussion

Since endogenous neurogenesis plays an important role in post-stroke functional recovery^25^, enhancing the proliferation, migratory, and differentiation capacity of NSPCs may benefit stroke survivors via promoting neurogenesis. We have previously demonstrated that medium B, a combination of EGF^26^ and fibronectin, could promote the proliferation and migration of cultured NSPCs^13^. In this study, we tried to extend our findings by investigating the effect of medium B on NSPCs using post-OGD cultured neurons (an *in vitro* stroke model) and MCAO rats (an *in vivo* stroke model). We found that medium B treatment enhanced the proliferation and migratory capacity of cultured NSPCs on the 3-dimentional structures and endogenous NSPCs of MCAO rats. Medium B also promoted neuronal differentiation of NSPCs in MCAO rats. In addition, medium B treatment diminished apoptosis in post-OGD cultured neurons with existence of NSPCs and in the infarct brain of MCAO rats. Subsequently, MCAO rats with medium B treatment showed more neuronal density in the infarct brain, better functional performance, and smaller infarcted volume than untreated MCAO rats, probably through the mechanisms of enhanced post-stroke neurogenesis and anti-apoptosis.

Using various 3-dimentional structures, we reproduced our previous findings that medium B promoted NSPC proliferation and migration. With medium B treatment, NSPCs can well attach to the chitosan scaffold and decellularized brain structures, both proliferating and migrating. In MCAO rats, continuous intraventricular infusion of medium B for about 7 days increased the number of Ki-67 or nestin-immunoreactive cells around the ipsilateral SVZ, indicating enhanced proliferation of endogenous NSPCs. In addition, neuroblasts are immature neurons, which in part differentiate from NSPCs in the SVZ or SGZ of the adult brain^27–30^. Medium B treatment promoted the migratory capacity of NSPCs in MCAO rats, noted by the longer migratory distance of neuroblasts from the SVZ than in untreated rats. As a result, medium B-treated MCAO rats also showed more Ki-67- or nestin-immunoreactive cells in both the striatum and cortex, representing high post-MCAO proliferation and migratory capacity of NSPCs. Previous studies have demonstrated that wnt signaling pathway (akt/GSK-3β/β-catenin) is crucial for neurogenesis^31,32^. After medium B treatment, we found that the levels of phospho-Akt, phospho-GSK-3β, and β-catenin were all higher in medium B-treated than in untreated MCAO rats, which may contribute to enhancement of NSC proliferation, migration and differentiation in our MCAO rats.

In the presence of NSPCs, medium B reduced neuronal apoptosis with high neuronal viability in both direct and indirect (Transwell) co-culture systems. Again, treatment with only EGF or fibronectin did not show similar effects. Notably, co-culture with higher amounts of NSPCs but not medium B treatment also did not show a benefit to post-OGD neurons, indicating that the anti-apoptotic effect of medium B was unlikely primarily via the enhancement of NSPC proliferation, but through triggering NSPCs to produce certain factors to diminish neuronal apoptosis. In both cultured neurons and infarct brain, we also detected a lower percentage of TUNEL-positive neurons, lower levels of cleaved caspase 3 (an apoptotic factor), higher levels of Bcl-xL and Bcl-2 (anti-apoptotic factors), and higher levels of phospho-Akt, phospho-GSK-3β, and β-catenin (upstream factors of anti-apoptotic pathways)^33–35^. Therefore, in addition to promoting NSPC neurogenesis, medium B also reduced neuronal death by enhancing the neuroprotective effect of NSPCs.

To investigate the neuroprotective factors secreted by NSPCs after medium B treatment, we performed a protein array analysis (RayBiotech, Norcross, GA) using the samples of culture medium, collected from the post-OGD neuronal culture with or without different treatments. Among the 90 analytic factors, interleukin-6 (IL-6), granulocyte macrophage-colony stimulating factor (GM-CSF), and tissue inhibitor of metalloproteinase-1 (TIMP-1) may contribute to the medium B-associated neuroprotective effect, according to the protein expression patterns (Supplemental Table S1) and the anti-apoptotic effects of these factors noted in previous studies^36–38^. The exact neuronal survival-promoting factors released from NSPCs triggered by medium B are still unclear and required further investigation.

In MCAO mice, conditional ablation of neurogesis worsened post-stroke motor and cognitive function^10^. In patients with subarachnoid hemorrhage (another stroke subtype), the presence of more proliferation-promoting factors in the cerebrospinal fluid was associated with better functional outcomes^39^. Therefore, endogenous neurogenesis plays an essential role in post-stroke recovery. In addition, upon cerebral ischemia, neurons may die through acute necrosis or subacute apoptosis^40,41^. Since neuronal necrosis is essentially irreversible, enhancing or supporting anti-apoptosis may be an important therapeutic strategy for post-stroke treatment to rescue damaged cells. In this study, medium B-treated MCAO rats showed higher neuronal density in the infarct brain, better functional performance, and lower infarcted volume than untreated rats, all of which were associated with greater post-MCAO neurogenesis and anti-apoptosis. Therefore, medium B likely enhanced post-MCAO neurogenesis and anti-apoptosis, resulting in more new generated neurons and less death of pre-existing neurons, with the net result of more neurons in the infarct brain with resulting better function recovery.

For different subtypes of stroke, current clinical management has been limited to thrombolysis/thrombectomy, hematoma evacuation, and prevention of stroke recurrence or stroke-related complications^2,42^. Until now, no other effective treatment for acute stroke exists, especially in terms of neuroprotection or neurogenesis^11^. We here demonstrated that intraventricular medium B treatment provided two therapeutic mechanisms: promoting endogenous neurogenesis and inhibiting neuronal apoptosis. This treatment strategy is thus worthy of application in future clinical trials in patients with acute ischemic stroke. In addition, patients with hemorrhagic stroke, including intracerebral hemorrhage or subarachnoid hemorrhage, may represent another attractive target for medium B treatment, considering that some of these patients would receive surgery for ventricular drainage^43,44^, which provides a direct route for intraventricular medium B infusion.

## Conclusions

This study extended our previous *in vitro* findings and demonstrated that medium B treatment enhanced the proliferation and migration of cultured NSPCs onto the 3-dimentional structures, and promoted post-MCAO NSPC proliferation, neuroblast migration, and neuronal differentiation in a rodent model of ischemic stroke. In addition, medium B treatment diminished neuronal apoptosis both in a co-culture system and in MCAO rats. Subsequently, post-MCAO intraventricular medium B infusion provided better functional recovery with smaller infarcted volume. Future clinical trials are anticipated to demonstrate the benefits of medium B treatment in patients with acute ischemic stroke.

## Supplementary information

Supplementary Information.

## Data Availability

The datasets generated during and/or analysed during the current study are available from the corresponding author on reasonable request.
