# Supplementary material for: Intraventricular Medium B Treatment Benefits an Ischemic Stroke Rodent Model via Enhancement of Neurogenesis and Anti-apoptosis
Source: Sci Rep. 2020 Apr 20;10:6596. doi: 10.1038/s41598-020-63598-0 (PMC7171187; doi:10.1038/s41598-020-63598-0)
Supplement: Supplementary file 1 — Supplementary Information. [file 41598_2020_63598_MOESM1_ESM.pdf]

Intraventricular Medium B Treatment Benefits an Ischemic Stroke Rodent Model via Enhancement of Neurogenesis and Anti-apoptosis

Yun-An Chen[1], Yi-Chieh Tsai [3],Yi-Dao Chen[2] , Der-Zen Liu[2], Tai-Horng Young[1,*], Li-Kai Tsai[3,*]

[1]Institute of Biomedical Engineering, College of Medicine and College of Engineering, National Taiwan University, Taipei, Taiwan

[2]Graduate Institute of Biomedical Materials and Tissue Engineering, College of Biomedical Engineering, Taipei Medical University, Taipei, Taiwan

[3]Department of Neurology and Stroke Center, National Taiwan University Hospital and National Taiwan University College of Medicine, Taipei, Taiwan

Correspondence should be addressed to:

*Li-Kai Tsai, M.D., Ph.D.; Department of Neurology, National Taiwan University Hospital, No. 7, Chung-Shan South Road, Taipei, 100, Taiwan

Tel: 886-2-23123456#23476; Fax: 886-2-2341-8395; E-mail:milikai@ntuh.gov.tw

*Tai-Horng Young, Ph.D.; Institute of Biomedical Engineering, College of Medicine and College of Engineering, National Taiwan University, Taipei, 100, Taiwan

Tel.: +886 2 23123456 ext 81455; Fax: +886 2 23940049; thyoung@ntu.edu.tw

Supplemental Methods

Three-dimensional structures

In the Transwell membrane study, neural stem/progenitor cells (NSPCs) were added in the upper part of Transwell plates (with 8 μm pore size) (Corning, NY) and medium B was added in the lower part. After culture for 7 days, the NSPCs on the upper side of the Transwell membrane were removed using a cotton swab. The NSPCs migrating through the pores and locating on the lower side of the membrane were then fixed with ice-cold methanol and glutaraldehyde for Hoechst 33258 (1:1000, diluted in PBS, Invitrogen, Carlsbad, CA) staining or fixed with 2.5% glutaraldehyde solution (Sigma-Aldrich, St. Louis, MO) for the scanning electron micrograph (SEM) experiments.

In the chitosan scaffold study, powdered chitosan (Sigma-Aldrich) was solubilized with 3% acetic acid in deionized water, frozen overnight at -20°C, and lyophilized in a freeze-dryer (Freeze Dryer-FD-series, PANCHUM, Taipei, Taiwan) under vacuum less than 50 Pa for 3 days. Residual acetic acid was removed from the lyophilized scaffold after three washes with NaOH solution and deionized water. The NSPCs were cultured on the scaffold for 7 days and then analyzed using immunocytochemistry or SEM.

In the decellularized brain study, the brain of a Wistar rat (rats 270-350 g) was washed in the deionized water and immersed in decellularized solution containing 1%

sodium dodecyl sulfate (SDS) and 0.05% EDTA in deionized water at room temperature. The decellularized solution was refreshed every 24 hours. After 3-4 days, the decellularized brains were placed in 1% antibiotic-antimycotic (Gibco, Pascagoula, MS) and 0.05% EDTA (Sigma-Aldrich) in PBS and the solution was refreshed twice a day for 3-4 days. All decellularizations were performed using agitated baths at 100 rpm at room temperature for 8 days. The NSPCs were cultured in the decellularized brains for 7 days and then analyzed using immunocytochemistry or SEM.

Immunocytochemistry and immunohistochemistry

In immunocytochemical characterization, cells were fixed with ice-cold methanol for 20 min, blocked with 1% bovine serum albumin (BSA) for 30 min at room temperature, and then incubated with rabbit anti-Ki67 polyclonal antibody (a proliferation marker; 1:300, Abcam, Cambridge, MA), mouse anti-nestin monoclonal antibody (a neural stem cell marker; 1:300, Merck Millipore, Billerica, MA), and mouse anti-MAP2 polyclonal antibody (a mature neurons marker; 1:200, Merck Millipore) overnight at 4°C. After wash, cells were incubated with Cy3-conjugated donkey anti-mouse IgG (1:200; Jackson ImmunoResearch, West Grove, PA), Alexa Fluor 488-conjugated goat anti-rabbit IgG (1:200; Jackson ImmunoResearch) and Hoechst 33258 (1:1000) for 2 hours at room temperature. The immunostained cells were visualized by

fluorescent microscope (Leica Microsystems DMI600, Wetzlar, Germany).

Immunohistochemistry was performed on cryostat brain sections that were collected from rats 15 days after MCAO with intracardiac formaldehyde perfusion (N=6 for each group). A series of 16 μm-thick sections were fixed with ice-cold methanol for 5 min, permeabilized with 1% Triton X100-PBS for 30 min, blocked with 3% fetal bovine serum in 0.1% Triton X100-PBS at room temperature for 2 hours, incubated with primary antibodies in blocking solution at 4°C overnight, and incubated with secondary antibodies and Hoechst 33258 (1:1000) for 2 hours at room temperature. The primary antibodies included rabbit anti-Ki67 polyclonal antibody (1:200 or 1:300, Abcam), mouse anti-nestin monoclonal antibody (1:200 or 1:300, Merck Millipore), mouse anti-MAP2 (microtubule-associated protein 2) polyclonal antibody (1:200), rabbit anti-glial fibrillary acidic protein (GFAP) polyclonal antibody (1:500, Abcam), mouse anti-GFAP monoclonal antibody (1:200, Sigma-Aldrich), rabbit anti-doublecortin (DCX) polyclonal antibody (1:500, Abcam), and rabbit NeuN monoclonal antibody conjugate alexa Fluor 488 (1:50, Abcam). The secondary antibodies included Alexa Fluor 488-conjugated goat anti-rabbit IgG, Cy3-conjugated donkey anti-mouse IgG, Alexa Fluor 488-conjugated goat anti-mouse IgG, and Cy3-conjugated donkey anti-rabbit IgG (1:200; Jackson ImmunoResearch) and Alexa Fluor 594-conjugated donkey anti-mouse IgG (Invitrogen). The immunostained samples were visualized by

confocal LSM880 microscope (Carl Zeiss AG, Oberkochen, Germany) with an optical thickness of 2 μm. The area with the immunoreactive signal was determined automatically using MetaMorph software (Leica Microsystems GmbH). Immunochemical detection of the Ki67 nuclear protein has been widely used to identify cells in the late G1 through M phases of the cell cycle for studying cell proliferation, including NSPC proliferation[1-3].

Western Blot

Samples of cultured neurons or brain tissues were lysed in RIPA (radio-immune precipitation assay) buffer (Roche Diagnostics, Basel, Switzerland) containing protease and phosphatase inhibitors. Equal amounts of protein from each sample were analyzed by sodium dodecyl sulfate-polyacrylamide gel electrophoresis (SDS-PAGE). Protein was transferred onto 0.22 μm polyvinylidene fluoride (PVDF) membranes (Millipore), blocked in 5% BSA-TBST (Tris buffered saline with Tween 20) buffer for 30 min and incubated with the primary antibodies at 4°C overnight and horseradish peroxidase - conjugated secondary antibodies (1:5000; Abcam) at room temperature for 1 hour. The primary antibodies included anti-Bcl-xL (1:1000, Cell Signaling Technology, Danvers, MA), anti-Bcl-2 (1:1000, Cell Signaling Technology), anti-Bax (1:2500, BD), anti-cleaved caspase-3 (1:1000, Cell Signaling Technology), anti-Akt (1:1000, Cell

Signaling Technology), anti-phospho-Akt$^{(Ser473)}$ (p-Akt, 1:1000, Cell Signaling Technology), anti-GSK-3β (1:2500, BD, Franklin Lakes, NJ), anti-phospho-GSK-3β$^{(Ser9)}$ (1:1000, Cell Signaling technology), anti-β-catenin (1:4000, Abcam), and anti-GAPDH antibodies (1:5000; Abcam). The Western blotting signals were then detected using an enhanced chemiluminescence (ECL; Millipore), acquired with the UVP BioSpectrum 810, and analyzed with Vision Works LS software (UVP, LLC, Upland, CA) (N=4 for each group).

Behavior testing

For the rotarod test, rats were placed on an accelerating rotarod cylinder (Rota-rod treadmills, Ugo Basile Biological Research, Ithaca, NY), in which the speed was slowly increased over 5 min from 5 to 80 rpm. The time that animals remained on the rotarod was measured. Each test consisted of four independent measurements, and the longest time spent on the device was recorded. The neurological severity score test was a composite of the nine subtests of motor performance (flexion of forelimb or hind-limb, head movement 10 degrees to the vertical axis, inability to walk straight, circling towards the paralytic side, falling down to the paretic side, immobility/staring, tremor, and seizures), two sensation subtests (visual and tactile placement and a proprioceptive test), and three reflex subtests (pinna, corneal, and startle reflex), grading 0 to 14

(normal score 0, maximal deficit score 14)[4]. In the body asymmetry test, rats were examined for lateral movements/turning when their bodies were suspended by the tail 200 mm above the testing table in 5 seconds. The number of initial head or upper body turns was counted in 20 consecutive trials (N=10 for each group). The rats receiving MCAO surgery with the neurological severity score of 4-7 on the second day were randomized into different groups for further studies without exclusion of rats after random assignment into the study groups. Rats were housed in propylene cages in animal housing facilities (National Taiwan University College of Medicine, Laboratory Animal Center) with each cage for maximal three animals, ambient temperature set at 25 ± 2°C, and humidity of 45–55%. Rats were maintained on a 12-h light/dark cycle with ad libitum access to pellet chow and water.

Protein array

We used the semiquantitative protein L-series antibody array chips, Rat L90 (RayBiotech, Norcross, GA) to investigate various neuroprotective factors secreted by NSPCs after medium B treatment. Ninety proteins were analyzed based on biotin labeling and laser fluorescence scanning techniques. We performed a protein array analysis using the samples of culture medium, collected from the post-OGD neuronal culture without or with different treatments. Hybridization proceeded according to the

manufacture of Rat L90. The signal fluorescence images were scanned by an Axon GenePix (Molecular Devices, Orleans, USA). The data was analyzed with GenePix Pro software (Molecular Devices).

References

1 Kee, N., Sivalingam, S., Boonstra, R. & Wojtowicz, J. M. The utility of Ki-67 and BrdU as proliferative markers of adult neurogenesis. *Journal of neuroscience methods* **115**, 97-105 (2002).

2 Harms, C. *et al.* Differential mechanisms of neuroprotection by 17 beta-estradiol in apoptotic versus necrotic neurodegeneration. *The Journal of neuroscience : the official journal of the Society for Neuroscience* **21**, 2600-2609 (2001).

3 Del Bigio, M. R. Proliferative status of cells in adult human dentate gyrus. *Microscopy research and technique* **45**, 353-358, doi:10.1002/(sici)1097-0029(19990615)45:6<353::aid-jemt3>3.0.co;2-m (1999).

4 Li, Y. *et al.* Intrastriatal transplantation of bone marrow nonhematopoietic cells improves functional recovery after stroke in adult mice. *Journal of cerebral blood flow and metabolism : official journal of the International Society of Cerebral Blood Flow and Metabolism* **20**, 1311-1319, doi:10.1097/00004647-200009000-00006 (2000).

Supplemental Figures

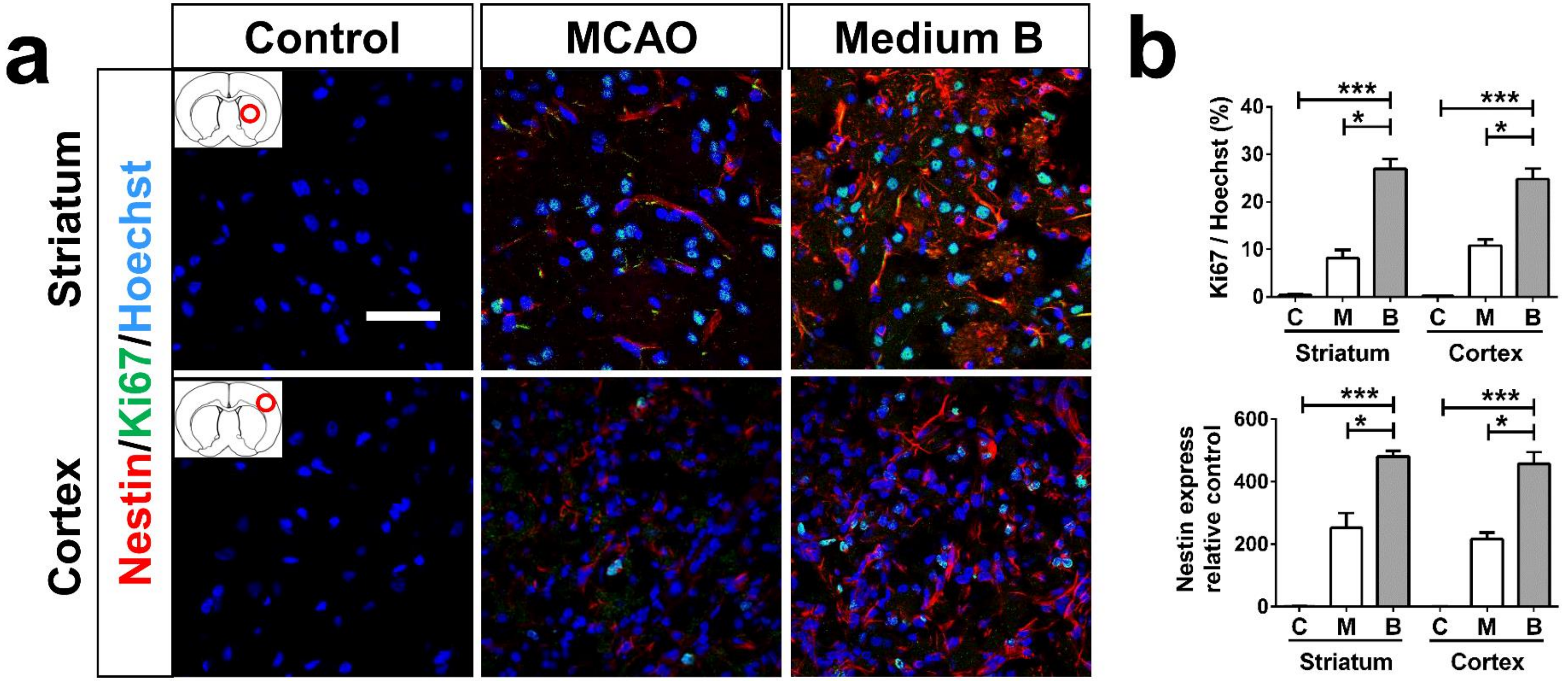

Supplemental Fig. S1 The effect of medium B on neural stem/progenitor cell (NSPC) proliferation in the striatum and cortex of rats with middle cerebral artery occlusion (MCAO). (**a**) Immunostaining with the proliferation marker Ki-67 (green), NSPC marker nestin (red), and nucleus marker Hoechst 33258 (blue) in the striatum and cortex of MCAO rats with and without medium B treatment and sham control rats is shown. (**b**) The proliferation capacity of NSPC is presented as the percentages of Ki-67 immunoreactive cells among Hoechst positive cells or intensity of nestin immunoreactive signals relative to control. N=6 for each group. Scale bar = 50 μm. *, $p<0.05$; ***, $p<0.001$. C, control; M, MCAO; B, medium B.

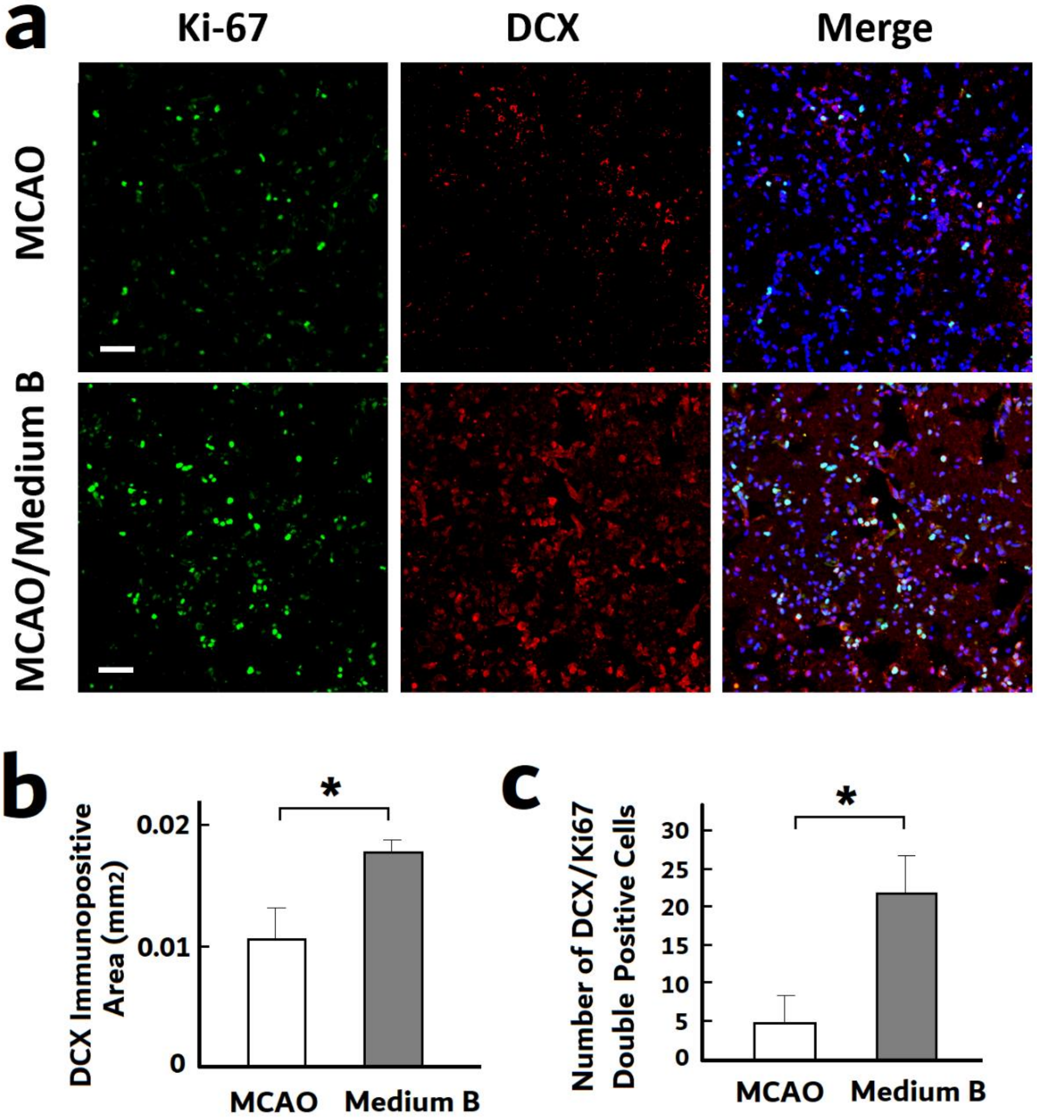

Supplemental Fig. S2 The effect of medium B on neuroblast in the striatum of rats with middle cerebral artery occlusion (MCAO). (**a**) Immunostaining with the proliferation marker Ki-67 (green), neuroblast marker doublecortin (DCX, red), and nucleus marker Hoechst 33258 (blue) in the striatum of MCAO rats with and without medium B treatment is shown. (**b**) The number of neuroblast is presented as the area of DCX immunoreactive cells per 0.2 mm^2. (**c**) The number of proliferative neuroblast is presented as the number of DCX and Ki-67 double immunoreactive cells per 0.2 mm^2. N=3 for each group. Scale bar = 50 μm. *, $p<0.05$.

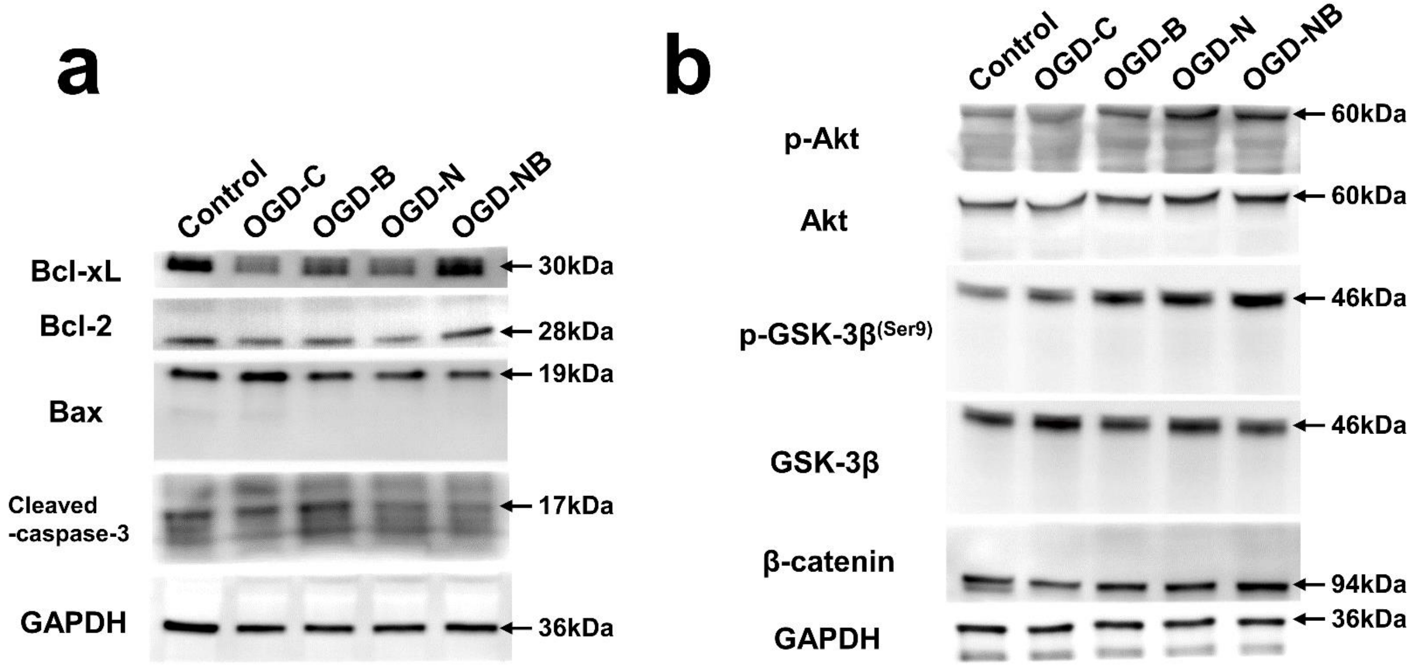

Supplemental Fig. S3 (a) Full scan blots from Figure 3b for Bcl-xL, Bcl-2, Bax and cleaved caspase-3, **(b)** Full scan blots from Figure 3c for Akt, phospho-Akt(Ser473), anti-GSK-3β, phospho-GSK-3β(Ser9), β-catenin and GAPDH.

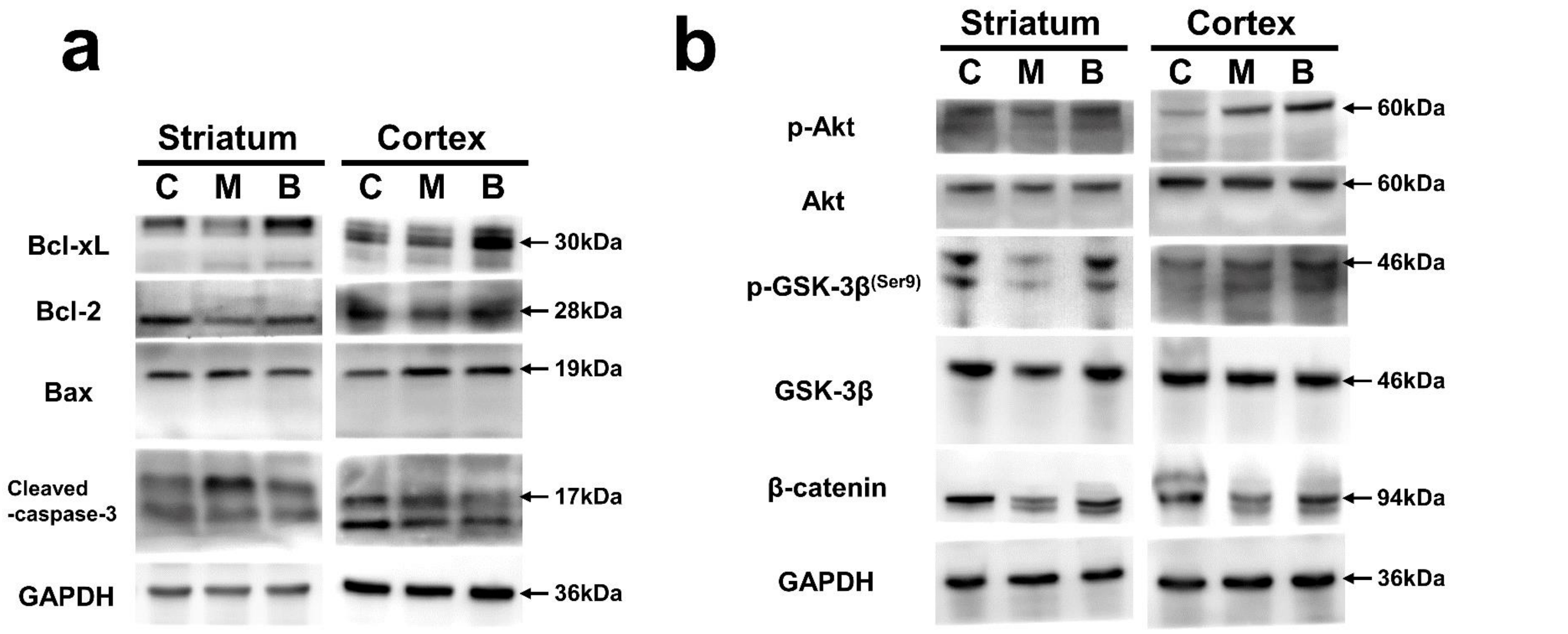

Supplemental Fig. S4 (a) Full scan blots from Figure 6a for Bcl-xL, Bcl-2, Bax and cleaved caspase-3, **(b)** Full scan blots from Figure 6c for Akt, phospho-Akt(Ser473), anti-GSK-3β, phospho-GSK-3β(Ser9), β-catenin and GAPDH.

Supplemental Table S1 Protein array analysis of the culture medium of neuronal culture with oxygen glucose deprivation (OGD).

Factor	OGD-C	OGD-B	OGD-N	OGD-NB	OGD-C	OGD-B	OGD-N	OGD-NB	
CNTF R alpha	205	331.1	288.6	471.7	1	1.61	1.41	2.30	*
IL-4	1965.5	3530.1	4681.6	4170.7	1	1.80	2.38	2.12	
MCP-1	4042	5307.6	5615.9	8168.6	1	1.31	1.39	2.02	*
VEGF	1677.5	2703.7	4603.9	3254.2	1	1.61	2.74	1.94	
CNTF	824.5	901.6	836.2	1598.6	1	1.09	1.01	1.94	*
IL-6	600	626.6	798.1	1152.9	1	1.04	1.33	1.92	*
Orexin A	1162.5	1123.4	2270.9	2188.8	1	0.97	1.95	1.88	
GM-CSF	2947.5	3752	4289.4	5526.4	1	1.27	1.46	1.87	*
TIMP-1	393.5	298	320.6	733	1	0.76	0.81	1.86	*
Neuropilin-2	2026.5	2718.2	3417.1	3716.8	1	1.34	1.69	1.83	
CD106	3377	3620.2	5791.2	6193.3	1	1.07	1.71	1.83	
Fractalkine	2565	3169	4240.6	4502.1	1	1.24	1.65	1.76	
Insulin	12423.5	13071.5	18165.5	21136.7	1	1.05	1.46	1.70	
IL-10	3943.5	3502	4557.6	6479.5	1	0.89	1.16	1.64	*
EGFR	1333	1756.5	1993.1	2134.4	1	1.32	1.50	1.60	

The culture medium of post-OGD neuronal culture without treatment (OGD-C) and post-OGD neurons with medium B (OGD-B), neural stem/progenitor cell (indirect, OGD-N) and combined medium B and neural stem/progenitor cells (NSPC) (indirect, OGD-NB) treatment was analyzed using a protein array kit (RayBiotech, Norcross, GA; AAR-BLG-1). Among 90 analytic proteins, only those with the expression ratio of the OGD-NB group over the OGD-C group greater than 1.5 times are shown.

* indicates a protein with the expression ratio of the OGD-NB group over the OGD-N group of greater than 1.2 times.

CD106 indicates cluster of differentiation 106; CNTF, ciliary neurotrophic factor; CNTF R alpha, ciliary neurotrophic factor receptor subunit alpha; EGFR, epidermal growth factor receptor; GM-CSF, granulocyte-macrophage colony-stimulating factor; IL-4, interleukin-4; IL-6, interleukin 6; IL-10, interleukin 10; MCP-1, monocyte chemoattractant protein-1; TIMP-1, tissue inhibitors of metalloproteinases-1; VEGF, vascular endothelial growth factor.
